# Anaemia is typical of pregnancies: capturing community perception and management of anaemia in pregnancy in Anambra State, Nigeria

**DOI:** 10.1186/s41043-016-0066-9

**Published:** 2016-08-31

**Authors:** Nkechi G. Onyeneho, Obianuju U. Igweonu

**Affiliations:** 1 Department of Sociology/Anthropology, University of Nigeria, Nsukka, Enugu State Nigeria; 2 Takemi Program in International Health, Department of Global Health and Population, Harvard TH Chan School of Public Health, 677 Huntington Avenue, Boston, MA USA; 3 Social Science Unit, School of General Studies, University of Nigeria, Nsukka, Enugu State Nigeria

**Keywords:** Anaemia in pregnancy, Attitude, Management, Nigeria, Perception

## Abstract

**Background:**

Anaemia during pregnancy continues to constitute significant challenge to maternal health in Nigeria and contributes substantially to the worsening maternal mortality ratio (MMR) in Nigeria despite a global reduction in MMR in response to effort to improve safe motherhood. The incidence of anaemia during pregnancy is still high (>40 %) in Nigeria, and attitudes and management practices are yet unclear as the peoples’ understanding of the phenomenon remains unclear. This study explored the perceptions/attitudes on anaemia during pregnancy and practices to prevent and/or manage it in Anambra State.

**Methods:**

In-depth interview and focus group discussion data were collected from health workers and mothers who delivered within 6 months preceding the study and from mothers and husbands of women who delivered within 6 months preceding the study, respectively.

**Results:**

The people expressed some knowledge of anaemia, being common in pregnancies. However, some expressed the view that anaemia being a typical sign of pregnancy cannot be prevented. Some mothers expressed desire for focused antenatal care services to control anaemia but lamented the attitude of the health workers, who make access to these interventions difficult.

**Conclusions:**

Control of anaemia in pregnancy should start with providing health education to pregnant women and their partners, who reinforce what the women are told during antenatal care, and with training health workers for friendlier attitudes to clients.

## Background

Anaemia, referred to as the most common hematologic abnormality, decreases the amount of oxygen reaching the tissues and organs of the body, thereby reducing their capacity to function [1, 2]. During pregnancy, such haemoglobin reduction has serious pregnancy-related complications, which could lead to maternal mortality. The two most common causes of anaemia in pregnancy (AIP) are iron deficiency and acute blood loss [3]. Iron requirements increase during pregnancy, and a failure to maintain sufficient levels of iron may result in adverse maternal-foetal consequences.

Anaemia is a global public health problem which has serious short- and long-term consequences during pregnancy and beyond [4]. Although anaemia can occur at any age and affect either gender, it is more prevalent in pregnant women and young children [5]. It leads to poor pregnancy outcomes and contributes to 20 % of deaths among pregnant women [6]. Untreated anaemia also leads to increased morbidity and mortality from these chronic conditions as well [4].

Global efforts to control anaemia appear futile especially in developing countries, where its devastating effects often make substantial impact on national economies. Beyond constituting 20 % of maternal death-specific cause, it is estimated that 58 % of pregnant women in developing countries are anaemic; and further to that, 50 % of all maternal deaths are linked to anaemia [7–9]. It is believed that half of all pregnant women in Africa are anaemic [10]. In fact, anaemia and malaria are the leading causes of morbidity and mortality in children in sub-Saharan Africa, Nigeria inclusive [11, 12]. Anaemia is considered as harmful and compelling as epidemics of infectious diseases, which generally affect all but the worst affected are infants, school-age children, and women of reproductive age [8, 13–16].

It is, however, preventable when access to supplements is guaranteed and when they are provided with minimum, consistent, and easily understandable information and counselling, indicating that these are key elements to ensure effective programmes [17]. Unfortunately, only 21 % of women took iron tablets daily for 90 or more days during their last pregnancy in Nigeria [18]. Some of the factors associated with compliance with the recommended regimen in preventing other medical conditions include patient factors such as ethnic origin, educational attainment, and knowledge and perceptions about the conditions and solutions [19–23]. Consequently, anaemia continues to constitute a major cause of maternal deaths despite efforts to promote positive pregnancy outcomes and maternal health in Nigeria [24]. In Nigeria, for instance, MMR increased from 545 to 575 deaths per 100,000 live births between 2008 and 2013 reflecting a worsening situation and failure in achieving the fifth target of the Millennium Development Goals (MDGs) [18, 25, 26].

Perception takes a central position in determining health-seeking behaviour [27]. People’s perceptions and judgement are often conditioned by assessing factors their traditions and culture consider important such as courtesy, responsiveness, attentiveness, and perceived competence of the health staff [28, 29]. According to the health belief models, response to health conditions depends largely on the perception of the severity of the health condition as well as susceptibility and efficacy of remedies [30]. This is critical in developing appropriate promotional messages and campaigns, aimed at creating demand for particular health interventions [31]. However, data on perceptions of health issues have generally been collected quantitatively with comparatively low reliance on qualitative methods of inquiry [32–39].

The effectiveness of AIP interventions, as is the case of all health interventions, does not only rely on their clinical efficacy but also on a range of social and cultural factors. The social and cultural factors are better captured in great detail by listening to the voices of the people using qualitative study tools. Attitudes and behaviours towards interventions are often shaped by social and cultural factors, and such factors are particularly relevant to the demand and utilization of AIP and influence how, where, and when pregnant women seek AIP prevention and treatment. Listening to the people express themselves freely will generate richer data set, which result from the probe facility of the qualitative study tools.

This study adopted the qualitative methods of inquiry in assessing the perceptions of people in Anambra State, Nigeria, on anaemia and how it influences their practices to prevent and manage AIP.

## Methods

### Study design

The study was exploratory and adopted a cross-sectional approach using qualitative methods of inquiry, based on in-depth interviews and focus group discussion designs to allow a description and analysis of perceptions/attitudes towards AIP and its management and prevention practices in Anambra State, Nigeria.

### Study area

The study was located in Onitsha South and Idemili South, two of the 21 local government areas (LGAs) in Anambra State, Southeast Nigeria, representing the urban and rural LGAs, respectively. The 2006 Nigeria population and housing census put the population of these LGAs at 136,662 and 207,683, respectively. Fegge town, in Onitsha South LGA, is an urban community made up of people from different walks of life. The town is heterogeneous and has a high population density (density 13,719.1/km^2^). On the other hand, Idemili South LGA consists of seven communities. These are largely rural communities. Each community has one PHC while the LGA has only one general hospital in Nnobi.

The selection of urban and rural LGAs was to ensure comparative data for this study. The major source of income for the people in the urban area is commerce and civil service while agriculture is the major source of income for the rural LGA.

### Study population and sampling

The target population for this study was women within the child-bearing age of 15–49 years who gave birth within 6 months before the survey. Their mothers and husbands were also covered in the study. Healthcare providers were also interviewed. The distribution of data collected in each local government area is contained in Table 1.

**Table 1:** Distribution of sample by LGA and groups of study participants

Groups	Local government areas (LGAs)					
	Rural		Urban		Total	
	Idemili South		Onitsha South			
	FGD	IDI	FGD	IDI	FGD	IDI
Mothers of women who delivered within 6 months before study	4		4		8	
Husbands of women who delivered within 6 months before study	4		4		8	
Health workers		4		4		8
Women who delivered within 6 months before study		6		6		12
Total	8	10	8	10	16	20

A total of six communities were randomly selected from each LGA. The focus group discussions were equally distributed across local government areas. This gave a total of 16 focus group discussions (FGDs) with husbands and mothers of women (15–49 years), who delivered within 6 months before the survey, and 20 in-depth interviews (IDIs) with health workers and women (15–49 years), who delivered within 6 months before the survey, in the entire study (Table 1). Each focus group discussion session consisted of eight to ten participants, selected with the convenience sampling technique, after receiving community consent following social mobilization.

Groups were selected according to participants’ availability. Women who delivered in less than 6 months to the study were equally included in the study based on their availability and willingness to participate in the study. The health workers, on the other hand, were selected through a two-stage sampling. First, eight health facilities were randomly selected from the list of health facilities in the study LGAs. The officer-in-charge of the selected health facility was purposively selected for the study.

#### Inclusion of subjects

As mentioned earlier, women of child-bearing ages (15–49 years) who delivered within 6 months before the study in the communities constituted the index population for the study. Their mothers and husbands who may belong to older age ranges were also included. At a central point in each community enlisted for the study, a route was randomly identified and the eligible women (15–49 years) were identified by the research assistants who went house to house following the randomly selected route. The Igbo people have a practice where a woman who delivered newly is nursed for a period by the mother (grandmother of the child). The practice is called “omugợ”. Some of these women, however, had other relatives, including young girls and house-helps, instead of their mothers playing the role. So only those with their mothers (grandmothers of the new babies) were included in the study. Similarly, those who did not have their mothers but have their husbands were included in the study. These grandmothers and fathers of the new babies were invited to participate in the FGD sessions. Participation was voluntary and on availability and convenience. So only those who came were included in the FGD session. For the in-depth interviews, focus was on the women who delivered within 6 months before the study. However, only such women who did not have their mothers or husbands in the community at the time of the study were enlisted. This was aimed at ensuring that households from where an FGD participant was drawn were not included in the IDIs, to avoid contamination of data. Contamination of data will occur when a participant in the study is given a second opportunity to rethink a response to an issue in the study and changes from the position held in the previous interview in the study. These women were requested to participate in the IDIs. The officers-in-charge of the health facilities in the study communities were purposively selected for IDIs.

### Instrument and method of data collection

The instruments for data collection were focus group discussion and in-depth interview guides. The discussions were tape recorded, where permission was granted. The instruments were pre-tested in one urban and two rural communities in Idemili North LGA, for the sensitivity of the instrument. These tools were able to elicit appropriate responses to the questions and issues raised in the IDIs and FGDs, respectively. The pre-testing also provided opportunity for giving targeted orientation on the methods and objectives of the study to the data collectors.

#### Data management and analysis

After reviews and corrections, all interview and discussion transcripts were typed with Microsoft Word processing package and converted into American Standard Code for Information Interchange Rich Text Format (RTF) files. These were coded and sorted using the Atlas.ti version 6 program.

Analysis placed emphasis on the interpretation, description, and recording/writing of what was actually said. The transcripts were first done in the local language and translated into English. In going through the transcriptions, phrases with contextual or special connotations were noted and pulled out as illustrative quotes.

## Results

### Social demographic characteristics of the respondents

A total of 171 persons (*N* = 171) were enlisted in the study and participated in the discussions (*n* = 151) and IDIs (*n* = 20). Respondents for the IDIs were mainly 8 health workers and 12 women who delivered in less than 6 months prior to the study. Four women who delivered 6 months prior to the study were interviewed in each LGA. On the other hand, six health workers were selected and interviewed in each of the LGAs (Table 1). Approximately half of the 151 participants in the FGD sessions were drawn from each of the participating LGA (Table 2).

**Table 2:** Distribution of FGD participants by socio-demographic characteristics (n-151)

Socio-demographic characteristics	Frequency	Percentage
LGA		
Idemili South	70	46.4
Onitsha South	81	53.6
Groups		
Mothers of women who delivered 6 months before study	79	52
Husbands of women who delivered 6 months before study	72	48
Marital status		
Married	147	97.4
Widowed	4	2.6
Educational level		
No formal education	6	4
Primary	55	36.4
Secondary	75	49.7
Post-secondary	15	9.9
Occupation		
Unemployed	42	28
Civil servant	44	29
Trader	45	30
Artisan	20	13
Age group		
35-39	24	15.9
40-44	29	19.2
45-49	36	23.8
50-54	26	17.2
55+	36	23.8

The health workers were mainly females with ages between 28 and 45 years. The women who delivered in less than 6 months before the study were aged between 20 and 31 years and were into civil service and trading.

Approximately half of the FGD participants fell into the two categories of FGD participants, namely mothers and husbands of women who delivered less than 6 months before the study (see Table 2). The participants were aged between 35 and 59 years with a mean age of 35.8 years (35.8 ± 7.9 SD). A total of 79 participants were females. One hundred and forty-seven of the respondents were married, and four were widows. Ninety of the respondents had at least secondary education.

The FGD participants were drawn from various occupational categories and were fairly evenly distributed with the exception of the group of artisans (13.0 %). Twenty-eight percent (28 %) was unemployed while 30 and 29 % were traders and civil servants, respectively.

### Knowledge of anaemia in pregnancy

The commonest problem mentioned was bleeding during pregnancy. Others were severe weakness, water breaking without labour, high fever and loss of consciousness during pregnancy, and swollen body parts among others. Asked if they are aware of anaemia, many of the urban participants indicated awareness.

The participants listed causes of AIP revealing varying degrees of knowledge between the urban and rural respondents. For instance, carrying twins or more babies was very popular among the rural respondents. Similarly, birth at short intervals was more commonly mentioned in the rural communities. On the other hand, such other causes as internal bleeding or bleeding from the virginal and diet low in blood-giving food were more commonly mentioned in the urban areas. Such other causes like inability to retain essential food nutrients were commonly mentioned in both the rural and urban communities. They did not recognise the role of blood-building abilities as a factor AIP.

There is a common belief among some women that anaemia is caused by exposure to evil forces. A few blamed it on eating foreign foods and not foods eaten by the older generations during pregnancy. They stressed that there are traditional foods meant for women during pregnancy. These were captured from the opinion of older mothers in FGD sessions held in both the urban and rural communities.

In Idemili South, FGD session for older mothers, different perceptions on causes of anaemia during pregnancy were put forward

*…anaemia is common, not just among pregnant women but among all the people here because of the harsh weather our people live in. At times in the morning when the weather should be cool, the sun rises with so much intensity and if you stay outside then for a long time, you will get anaemia.* [Participant: FGD, older mother; Onitsha South]

Other participants in the FGD sessions as well as the health workers blamed it on lifestyles and feeding habits of the women. They noted that pregnant women need blood for both the mother and child, and this means greater nutritional requirements. Here are samples of some of such comments:

*Anaemia results from poor dietary intake during pregnancy to meet the nutritional requirements of the child and mother. … Most of these women either do not know or do not have access to such food items that will help them build their blood. They eat mainly tuber carbohydrates like yam and cassava.* [Respondent: IDI, Health worker; Onitsha South]

With respect to effects of AIP, still birth was frequently mentioned in every community. Some mentioned other effects to include premature birth and retardation of the growth of the babies born in that condition. Participants displayed ignorance of other dangers like the occurrence of angina (pain in the left side of the woman’s chest) among others, even when raised as items for discussion.

Some of the participants in the FGDs and respondents to the in-depth interviews, with the exception of the health workers indicated that anaemia cannot be prevented as it is a typical sign of pregnancy. The following quotes illustrate this.

*Anemia is a typical sign of pregnancy because it shows that the woman’s body is working for two people* [Participant: FGD, older mothers, Idemili South]

***…****there is nothing like prevention of blood shortage other than replacing it. I don’t believe shortage of blood can be prevented. The things that suck the blood cannot be seen. Sometimes they are spiritual and how do you deal with spirits. So I don’t think anaemia can be prevented.* [Participant: FGD, older mothers; Idemili South]

However, the male participants in an FGD session in Fegge community stressed that blood is something you gain or lose depending on your lifestyle. According to one of the participants,

*Shortage of blood in the body or what you call anaemia can be prevented. The quantity or quality of blood in somebody’s body has to do with what the person eats or drinks. A pregnant woman should ensure that the blood in her body is enough to support her and the baby in her stomach by eating good food and taking blood tonic always.* [Participant: FGD, male; Onitsha South]

The health workers confirmed that most women who attend antenatal clinics during their pregnancies are aware of AIP. According to one of the health workers interviewed in the health facility in Fegge,

*…awareness of anaemia in pregnancy in a basic knowledge the people are given in the clinic during antenatal care seminars… so they should know the signs and preventives.*

The participants indicated varied ways of preventing AIP to include food habits and dieting, proper medication, and food supplementation as well as regular checkups in health facilities. They mentioned daily intake of fruits as a way of preventing AIP. Other strategies, among the urban respondents, include intake of fairly cooked vegetables and supplementation of diets with iron tablets.

With respect to managing AIP, the participants mentioned that a woman with AIP should report to a health worker for proper care. Others mentioned food supplementation with folic acid and identification and treatment of the cause of the condition. However, the health workers lamented that women come to hospital late in their pregnancy. For example,

*…most of them end up coming to the hospital but first of all, they try local concoctions and herbs which they believe to be more effective than western medicines. Upon the persistence of the symptoms they go to patent medicine dealers who mix all sorts of drugs for them. The pattern goes from herbs to patent medicine dealers then to the hospital when they are dying….* [Respondent: IDI, health worker; Idemili South]

Also, the FGD session with husbands of pregnant women in Onitsha threw more light on how their wives seek treatment when they suspect anaemia.

***…****when she is sick, we go to chemist first and buy some drugs…. If that doesn’t work, we’ll now go to Nurse (auxiliary nurse) for more treatments, when we have tried all these ones, we will now go to hospital.* [Participant: FGD, Adult male; Onitsha South]

### Antenatal-care-seeking behaviour during the last pregnancy

In the urban communities, respondents indicated that they received antenatal care during their last pregnancy. Some did not in the rural communities.

Those who used antenatal care services made their first visits at various stages of their pregnancies; some in the first month of their pregnancies while others waited until the sixth month of the pregnancy to seek antenatal care services. Older women and husbands in the rural and urban communities stressed as follows:

*… pregnant women like to go for antenatal but the problem is the quality of care they get. The health workers just shout at the pregnant women. When you complain of an ailment, they give you anything and argue that the drugs are out of stock. The private hospitals are better but are too money-focused.* [Participant: FGD, older mother; Idemili South]

***…****my son’s wife goes to one private hospital for antenatal because the first time she went to the government hospital, she spent the whole day, yet she was not attended to. These private clinics do not give necessary health education.* [Participant: FGD, older mother; Idemili South]

Some of the participants indicated that they experienced anaemia at different stages of their pregnancy.

## Discussion

This study revealed different levels of awareness of AIP. Some perceive it as preventable and manageable while others argued to the contrary. The latter argues that anaemia is a normal occurrence in pregnancy. These findings confirm findings from other studies, which identified perception as a major driver of the people’s attitude towards the interventions. Those who hold this view argue that utilization of such health interventions such as focused antenatal care services is only partially explained by availability, while the major decider lies in the people’s perception, which forms basis of their decision on whether or not to use the interventions [9, 27, 28].

Some scholars had also argued that assessment of the people’s perceptions is germane to the development and delivery of appropriate health interventions [31]. This study indicated that pregnant women perceived anaemia as a normal sign of pregnancy. Consequently, their perception of the prevention is influenced by this attitude. They argue that there is no prevention for blood shortage, which must occur in every pregnancy. This is not peculiar to Nigeria. A study in Mumbai revealed that anaemia is perceived as normal during pregnancy [40].

Despite awareness of the causes and implication of anaemia in pregnancy, misconceptions and poor attitude of health workers towards patients affected the utilization of the focused antenatal care services for the prevention and management of anaemia during pregnancy. Focused antenatal care at health facilities is key in the delivery of anaemia in pregnancy prevention interventions [12, 16, 23, 33]. However, the women seemed discouraged with the health system and seek alternative health care instead [29]. Some studies have highlighted the social, cultural, and health system constraints to the use of health facilities [16]. Health workers’ attitudes and behaviour towards pregnant women and attitudes towards specific services offered can also potentially deter women from accessing ANC at health facilities [33, 41].

The limitation of this study is the inability to generalise the results to larger populations, being a qualitative study in which the inclusion of participants was based largely on availability and not on any rigorous statistical consideration. Randomization was only limited to the selected communities. Consideration was not given to ensure the inclusion of all necessary parameters of the larger population. Closely linked to this is the inability to make statistical conclusions on the occurrence of the perception. This was not the intention in this study. The main intention is to capture perceptions about anaemia in pregnancy and its management and prevention practices among the people of Anambra State, Nigeria. The study provides data that could form the basis of a study using a more rigorous statistical and mixed method design. It provides a range of perceptions that would inform categories to be used in developing structured data capture tool for quantification in future.

## Conclusions

The findings here confirm the arguments in the health belief model, and one can conclude that pregnant women in Anambra State would adopt anaemia preventive measures if these measures were available and if they are sufficiently educated on the values of these measures. However, the health workers who should educate and encourage the women to make optimal use of the focused antenatal care services are themselves short of world expected standards [41]. The health workers also need to be trained on appropriate communication skills that will ensure they play their roles as facilitators on the use of focused antenatal care services positioned to control anaemia during pregnancy. It is thus recommended that stakeholders ensure health education to promote effective access to the anaemia-in-pregnancy control services among pregnant women as well as target health workers to create a friendlier environment for the clients.

## Abbreviations

AIP: Anaemia in pregnancy
ANC: Antenatal care
FGD: Focus group discussion
IDI: In-depth interview
LGA: Local government area
MDGs: Millennium Development Goals
MMR: Maternal mortality ration
RTF: Rich Text Format
